# Conformal prediction quantifies wearable cuffless blood pressure with certainty

**DOI:** 10.1038/s41598-025-09580-0

**Published:** 2025-07-23

**Authors:** Zhan Shen, Tapabrata Chakraborti, Christopher R. S. Banerji, Xiaorong Ding

**Affiliations:** 1 School of Life Science and Technology, University of Electronic Science and Technology of China, Chengdu, 611731 China; 2 The Alan Turing Institute, London, UK; 3 UCL Cancer Institute, University College London, London, UK; 4 King’s College London, Comprehensive Cancer Centre, London, UK; 5 Yangtze Delta Region Institute (Huzhou), University of Electronic Science and Technology of China, Chengdu, 611731 China

**Keywords:** Ambulatory cuffless blood pressure measurement, Conformal prediction, Uncertainty quantification, Confidence interval, Hypertension, Diagnosis

## Abstract

Though wearable cuffless blood pressure (BP) measurement technology has attracted significant attention from both academia and industry, the ability of existing methods and devices to track dynamic BP changes and provide reliable BP readings remains low, especially in ambulatory environments. This study develops and validates an algorithm for 24-h ambulatory cuffless BP confidence intervals (CIs) estimation with conformal guaranteed coverage of the true BP values using wearable electrocardiogram (ECG) and photoplethysmogram (PPG) on subjects in the ambulatory setting. First, a quantile loss-based Gradient Boosting Regression Tree (GBRT) model was trained to obtain ambulatory BP estimates along with model uncertainty. The model uncertainty was then calibrated using conformal prediction to obtain CIs with guaranteed reference values coverage. Ambulatory physiological data from 483 participants from the Aurora-BP study dataset were used for model validation. For ambulatory measurements during the daytime phase, the mean absolute difference (MAD) of the systolic BP (SBP) and diastolic BP (DBP) estimated by the proposed model was 14.32 mmHg and 9.53 mmHg, respectively. For ambulatory measurements during the nighttime phase, the MAD of SBP and DBP estimated by the proposed model were 14.22 mmHg and 10.13 mmHg, respectively. Providing CIs with guaranteed reference BP coverage for 24-h ambulatory BP estimation can enhance the trust of patients and physicians in wearable devices, thereby facilitating the prevention, screening, and management of hypertension.

## Introduction

Hypertension, or high blood pressure (BP), is one of the most important risk factors globally for heart disease and stroke, causing 10.8 million deaths worldwide in 2019^1^. From 1990 to 2019, the number of adults aged 30-79 with hypertension doubled worldwide^2^. Worse still, for relevant low- and middle-income countries, only 10% of patients can keep their BP below the hypertension threshold^3^. Therefore, there are still great challenges in the detection, diagnosis, and management of hypertension worldwide.

Traditional BP measurement methods, such as commonly used oscillometric sphygmomanometers, can only measure BP intermittently, making it difficult to detect hidden hypertension and white-coat hypertension. Further, the pressurized inflated cuff may cause user discomfort and result in a low adherence rate due to its obtrusive nature^4^. Invasive arterial catheterization can provide continuous monitoring of BP, but requires professional operation and is only suitable for clinical scenarios^5^. In contrast, cuffless measurement techniques can be integrated into wearable devices (e.g., smartwatches or patches), offering continuous monitoring capabilities that significantly improve long-term user compliance^6,7^.

Cuffless BP monitoring technology is based on wearable sensors that can acquire cardiovascular signals (such as electrocardiogram (ECG), photoplethysmogram (PPG), and tonoarteriogram (TAG), etc.) for indirect BP estimation, which has attracted considerable attention across academia and industry. The traditional cuffless BP measurement methods indirectly estimate BP based on the Moens–Korteweg (M–K) equation^8^ (that is, $PWV = \sqrt{Eh/\rho D}$) and the correlation between arterial elasticity and BP values (that is, $E = E_0 e^{\gamma P}$). Currently, in the context of big data, combined with the powerful predictive capabilities of machine learning or deep learning models, it is expected that a more accurate and non-invasive BP estimation model can be established. However, most methods can only effectively predict BP in a controlled environment. This is because the data used to train the model only comes from physiological data collected when the subjects’ BP is relatively stable at rest, which may not work for subjects in an ambulatory setting. Moreover, the high variability of BP of subjects in an ambulatory environment and motion artifacts from daily activities will be the main source of uncertainty (e.g., epistemic uncertainty and aleatoric uncertainty) in the cuffless BP estimation model. These inherent uncertainties frequently induce clinically significant prediction errors in BP, substantially undermining confidence in measurement reliability among both patients and clinicians^9^.

Our previous work^10^ verified the effectiveness of confidence intervals (CIs) for BP prediction and hypertension risk warning by verifying data collected from patients in the intensive care unit (ICU). Critically ill patients in the ICU are generally immobilized under close monitoring, and their hemodynamic parameters (e.g., arterial pressure, cardiac output) remain intrinsically dynamic due to complex pathophysiology and therapeutic interventions. This contrasts with ambulatory settings where extrinsic factors–including postural shifts, metabolic demands from physical activity, and environmental stressors–induce more pronounced BP variability. Consequently, while continuous BP estimation in the ICU requires addressing endogenous biological fluctuations, ambulatory monitoring faces compounded challenges from both physiological variability and exogenous noise sources (motion artifacts, measurement site instability, and autonomic nervous system responses to daily activities). Therefore, obtaining reliable CIs for BP estimations in an ambulatory environment will be more challenging.

**Fig. 1:**
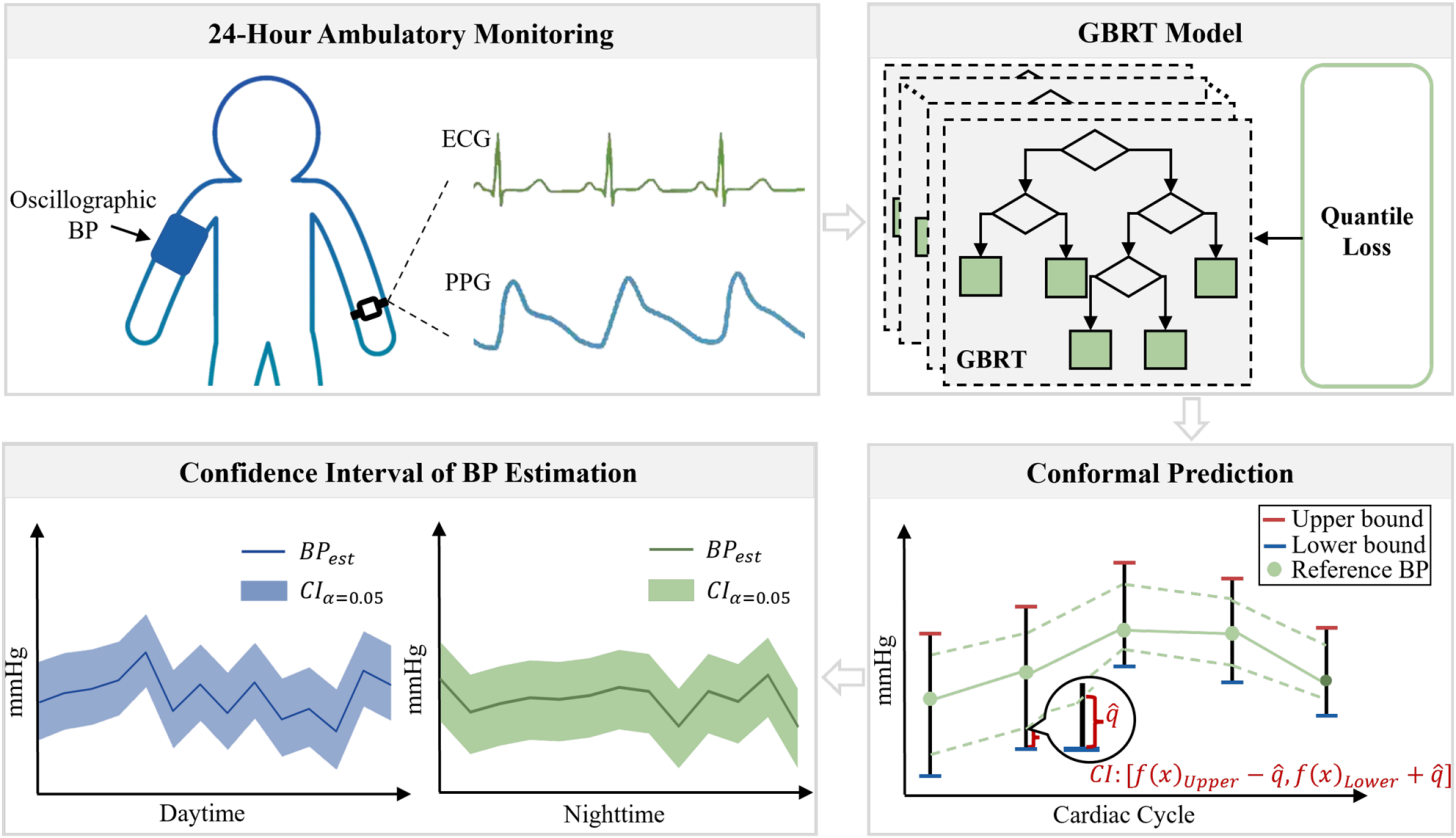
Dynamic workflow of the proposed ambulatory blood pressure (BP) measurement method.

In this study, ambulatory BP measurement was used to explore the problem of generating CIs for wearable cuffless BP measurements. Fig. 1 shows the workflow of the proposed method. In a 24-h ambulatory monitoring scenario, wearable devices are used to collect ECG and PPG signals, and an oscillographic device is used to collect reference BP signals. These physiological signals are then transferred to a machine learning model for training to obtain BP prediction values and the corresponding model uncertainty. The model uncertainty is then calibrated using conformal prediction to obtain CIs for guaranteed coverage of reference values. CIs can cover the reference BP value with a certain probability, thereby enhancing clinical trust in the BP estimation model and preventing potentially dangerous clinical decisions due to model failure. The key novelties and contributions of this study are as follows:

1. *Novel Cuffless BP Estimation Model with Uncertainty Quantification*: The study proposes a new method using a Gradient Boosting Regression Tree (GBRT) model combined with conformal prediction to estimate cuffless blood pressure (BP) and its confidence intervals. This approach provides reliable BP estimates with guaranteed coverage of true values, even in dynamic ambulatory settings.
2. *Enhanced Clinical Trust and Decision-Making*: By providing confidence intervals, the study aims to increase the trust of patients and physicians in wearable cuffless BP devices. The use of the incremental cost-effectiveness ratio (ICER) helps balance reliability and precision, supporting better clinical decision-making for hypertension management.
3. *Validation with Real-World Data*: The method is validated using a large-scale ambulatory dataset (Aurora-BP) with 483 participants. The model demonstrates effective tracking of dynamic BP changes during both daytime and nighttime, showing comparable performance and reliability in real-world conditions.

## Methods

### Study design and participant

The Aurora-BP dataset, a large-scale public dataset from Microsoft Research’s Aurora project^11,12^, was used to validate the proposed method. The dataset contained heterogeneous individuals who were balanced in terms of gender, age, and the condition of hypertension. The subjects were equipped with wrist-wearable devices to acquire synchronized ECG and PPG signals. Oscillometric BP was automatically and dynamically monitored (measurements were taken every 30 min during waking hours, and every 60 minutes at night) as the reference standard. Two separate protocols were implemented in the Aurora study - auscultatory and oscillometric. In the auscultatory protocol, data were measured manually by trained observers using an auscultatory sphygmomanometer during the subject’s initial and return in-lab visits (at least 24 hours between the two visits). In the oscillometric protocol, data were measured using an ambulatory blood pressure monitor (ABPM), which included measurements manually activated by the observer during the in-lab visit, as well as ambulatory measurements automatically triggered by a programmed schedule during the periods between in-lab visits.

**Fig. 2:**
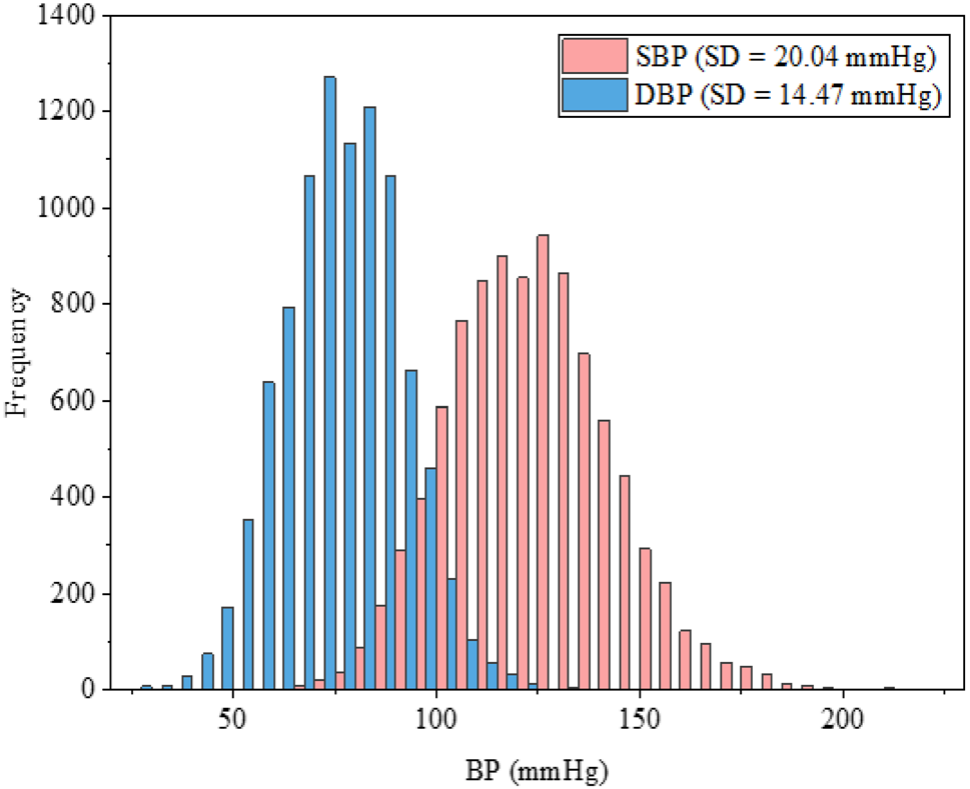
Distribution and standard deviation (SD) of systolic blood pressure (SBP) and diastolic blood pressure (DBP) of the identified subjects from the Aurora-BP dataset.

In this study, the oscillometric protocol data was used for model training and validation. The ambulatory dataset included 483 subjects with a mean age of 45.6 ± 9 years, whose height, weight, BP distribution, hypertension, and other cardiovascular diseases were measured. The subject’s characteristic information is shown in Table 1. During the experiment, each subject wore a wearable device containing a PPG sensor, a one-lead ECG sensor, and an epidermal pressure sensor to simultaneously acquire the PPG signal, the one-lead ECG signal, and the radial pressure signal. The subject wore an oscillometric BP measurement device (Spacelabs OnTrak 90227)^13^ on the left or right (randomized) brachial arm to measure reference BP values. Fig. 2 shows the distribution and standard deviation (SD) of SBP and DBP in the Aurora-BP dataset. The SD of DBP is significantly smaller than that of SBP, indicating that the changes of DBP in this dataset are relatively stable. For ambulatory measurements, data were collected every half an hour during the subjects’ approximate awake time (8 am–8 pm) and every hour during the night phase (8 pm–8 am) following the guidelines of the European Society of Hypertension^14^. The wearable signals and the reference BP were collected with a synchronized acquisition time clock.

**Table 1:** Basic information of subjects.

	All patients (n = 483)	Non-hypertension (n = 267)	Hypertension (n = 216)
Age, years (Q1–Q3)	46 (28–61)	44 (28–60)	48 (31–61)
Gender (Male), n(%)	248 (51%)	114 (43%)	134 (62%)
BMI, lb/in2 (Q1–Q3)	24 (12–59)	23 (12–50)	25 (14–59)
BP recordings, n	9386	5626	3760
SBP, mmHg (mean ± SD)	123.48 ± 20.04	119.70 ± 18.55	127.26 ± 20.81
DBP, mmHg (mean ± SD)	75.80 ± 14.47	73.66 ± 13.70	77.94 ± 14.96
*Medical history*			
Diabetes, n ($\%$)	55 (11%)	15 (6%)	40 (18%)
Arrythmia, n ($\%$)	10 (2%)	3	7 (3%)
Previous stroke, n ($\%$)	8 (2%)	3 (1%)	5 (2%)
Coronary artery disease, n ($\%$)	4 (1%)	0	4 (2%)
CVD meds, n ($\%$)	186 (38%)	21 (8%)	165 (76%)

### Data preprocessing

The Microsoft Research project team has made public the original physiological data and the physiological features extracted after preprocessing. This study directly selected and resampled the publicly available features and did not specifically involve the original data processing steps. However, the data preprocessing process was still briefly outlined to ensure the fluency and readability of the article. Microsoft researchers first used an algorithm to control the clock synchronization of physiological signal acquisition and oscillometric ABPM equipment and divided 15-s windows for subsequent analysis. In the data preprocessing part, Microsoft researchers used filters such as the DC block filter (0.1 Hz cutoff)^15^ and the lowpass 7th-order elliptical filter (40 Hz passband, 45 Hz stopband, 0.1 dB passband ripple)^16^ to process ECG and PPG signals to filter out baseline drift and high-frequency electromyographic interference contained therein. Then the Pan-Tompkins algorithm^17^ was used to identify the R peak of the ECG signal to ensure time alignment between different sensor devices. Finally, they evaluated the signal quality score based on signal artifacts, signal-to-noise ratio, and consistency between pulses in the window, and set a threshold to remove low-quality signals. In the feature extraction part, the research team at Microsoft implemented a comprehensive morphological analysis pipeline, extracting 14 distinct groups from synchronized multi-modal physiological signals. These features were derived from tonometric, PPG, and ECG signal segments captured within predefined analysis windows. Notably, the extracted features include clinically significant parameters such as: radial Pulse Arrival Time (rPAT), which serves as a proxy for vascular elasticity and peripheral resistance dynamics, and Heart rate (HR), a fundamental indicator of cardiac output and myocardial contractility performance. Then they took the median of multiple features in the same window as a representative. In this study, seven physiological features extracted from ECG and PPG signals were selected to train the BP estimation model. The selected features and their definitions are shown in Table 2.

In this study, the number of data samples affects the performance of model estimation during model training and conformal prediction calibration. To ensure data balance, this study downsampled the data during the daytime phase, using the first measurement as the representative of two measurements within an hour to ensure that each subject had the same data sampling rate during the daytime and nighttime phases. When the first measurement was invalid, the latter measurement was selected as the representative. In addition, blank data segments caused by acquisition failure, sensor detachment, etc., were filled with the previous data, ultimately ensuring that the sample size of the daytime and nighttime phases in the training set, calibration set, and test set was consistent.

**Table 2:** Definition of physiological features used for model estimation.

Feature	Definition
Arm Angle^12^	The angle of the arm relative to the reference line
Beat Length^12^	PPG signal cardiac cycle length
dP/dt^18^	PPG signal maximum slope point
Heart Rate^19^	The number of times the heart beats per minute
Heart Rate Variability^20^	Variability of the time intervals between consecutive heart beats
Quality^12^	PPG Signal Quality Score
rPAT^21^	Time delay between ECG waveform and radial artery waveform

### Cuffless BP Estimation Model

A gradient boosting regression tree (GBRT) based model^22^ combined with a customized quantile loss function was developed to estimate the cuffless BP and its confidence interval. GBRT is a machine learning method that uses boosting technology to improve traditional decision tree estimation. Its core idea is to aggregate a group of “weak” models to generate a single “strong” ensemble model. Compared with traditional regression models, GBRT’s boosting design helps deal with prediction situations with limited feature inputs and can capture the complex nonlinear characteristics of the prediction variables. Compared with neural network models, GBRT can be directly combined with quantile loss to generate CIs for BP predictions without modifying the network structure, thus having higher training stability^23^. The GBRT modeling process is as follows.

First, assume that the training set data is ${(x_i,y_i)}_{i=1}^N$, where $x_i$ represents the input feature, and $y_i$ represents the predicted target. The loss function can be defined as the square error, the mean absolute error (MAE), or Huber error. The square error is more susceptible to outliers, while MAE is more robust to outliers. In addition, compared with the Huber loss, MAE is simple to calculate and more interpretable when used for BP estimation. This study uses MAE as the loss function:

1 $$\begin{aligned} L(y,f_m(x))=\frac{1}{N}\sum _{i=1}^N\left| y_i-f_m(x_i)\right| \end{aligned}$$

where $f_m(x_i)$ is the model prediction value, *N* is the number of samples. Suppose that the GBRT model will build *M* decision trees and the GBRT framework starts with the model $f_0(x)$. For each iteration, $m=1,2,\ldots , M$, the least squares formula is used to solve the optimal hyperparameter $\alpha _m$:

2 $$\begin{aligned} \alpha _m=argmin_{\alpha ,\beta }\sum _{i=1}^N\left[ r_i-\beta h(x_i,\alpha )\right] ^2 \end{aligned}$$

where $\beta$ is the weight factor and $r_i$ is the negative gradient evaluated using the previous model:

3 $$\begin{aligned} r_i=-\left[ \frac{\partial L(y_i,f(x_i)}{\partial f(x_i)}\right] _{f(x)=f_{m-1}(x)}, i=1,2,\ldots ,N \end{aligned}$$

The weights or gradient descent steps of the resulting decision tree can be further optimized as a one-dimensional optimization problem:

4 $$\begin{aligned} \rho _m=argmin_{\rho }\sum _{i=1}^NL\left[ r_i,f_{m-1}(x_i)+\rho h(x_i;\alpha _m)\right] ^2 \end{aligned}$$

Finally, the newly evaluated residual model is added to the previous model, and the specified number of iterations is performed in sequence to obtain the final regression model:

5 $$\begin{aligned} f_m(x)=f_{m-1}(x)+\rho _mh(x;\alpha _m) \end{aligned}$$

In addition, this study combines the quantile loss^24^ with the GBRT model, expands the formula (1), and performs synchronous training. Quantile loss is a flexible and general statistical method that applies to various basic models, including linear regression, decision trees, neural networks, etc. Its core idea is to fit the conditional distribution of the target at different quantiles by minimizing the quantile loss function, to obtain the predicted values of different quantiles. Compared with the traditional mean square error loss function, the quantile loss function is more adaptable to outliers and skewed distribution data, so it shows better robustness and reliability when processing real scenarios such as medical data. The definition of quantile loss is as follows:

6 $$\begin{aligned} \begin{aligned} L(y,f_m(x))&=\frac{1}{N}\sum _{i=1}^NI_{f_m(x_i)\ge y_i}(1-\gamma )\left| y_i-f_m(x_i)\right| \\&\quad + I_{f_m(x_i)<y_i}\gamma \left| y_i-f_m(x_i)\right| \end{aligned} \end{aligned}$$

where $\gamma$ is the quantile level, its value usually between 0 and 1. Quantile loss mainly separates the overestimation ($f_m(x_i)\ge y_i$) and underestimation ($f_m(x_i)<y_i$) of the model, and assigns different coefficients to each. When $\gamma >0.5$, the loss caused by underestimation will be greater than the loss caused by overestimation; when $\gamma <0.5$, the loss caused by overestimation will be greater than the loss caused by underestimation. When $\gamma =0.5$, the quantile loss will become the mean absolute difference loss, consistent with the formula (1). In this study, three quantile losses $\gamma =0.05$, $\gamma =0.50$, and $\gamma =0.95$, were selected for GBRT training. When the quantiles $\gamma =0.05$ and $\gamma =0.95$ are set, the estimated values can be used as the upper and lower confidence limits of the model estimate, that is, $f(x_i)_{0.05}$ and $f(x_i)_{0.95}$. The model prediction value will be lower than $f(x_i)_{0.05}$ with a probability of 5% and higher than $f(x_i)_{0.95}$ with a probability of 5%. However, due to the lack of confidence guarantee of the fitted quantiles, it is necessary to combine conformal predictions to generate a statistically rigorous confidence interval.

### Confidence intervals generation

Conformal prediction is a statistical method that is mainly used to generate statistically rigorous confidence intervals for model predictions, which can cover reference values with a certain probability. The calibration dataset was used to perform conformal prediction to calibrate the confidence interval generated by the GBRT model. The prediction set obtained after implementing conformal prediction can be expressed as:

7 $$\begin{aligned} 1-\alpha \le P(y_{ref}\in \mathbb {C}(x_{cali})) \le 1-\alpha +\frac{1}{n+1} \end{aligned}$$

where $(x_{cali},y_{cali})$ is the calibration set sample point, and $\alpha$ is the user-selectable error rate level, *n* is the number of samples in the calibration set. The uncertainty interval $\mathbb {C}$ covers the uncertainty range of the model prediction and can ensure that the reference target, $y_{ref}$, is covered with a probability of $1-\alpha$. This study’s specific implementation process of conformal prediction is as follows.

After the GBRT model is trained with quantile loss, the predicted BP value and the upper and lower bounds can be calculated for the calibration set data, that is, the two quantiles $f(x_i)_{\alpha /2}$ and $f(x_i)_{1-\alpha /2}$. When implementing conformal quantile regression, the conformal score is first defined as the difference between the quantiles closest to the reference value $y_{ref}$:

8 $$\begin{aligned} s(x_i,y_i)=max\left[ f(x_i)_{\alpha /2}-y_i,y_i-f(x_i)_{1-\alpha /2}\right] \end{aligned}$$

When the error rate is set to $\alpha$, conformal prediction can ensure that the probability that the prediction set contains the reference value is exactly $1-\alpha$; $1-\alpha$ is also called the marginal coverage. Then $\hat{q}$ was defined as the $\lceil (n+1)(1-\alpha )\rceil /n$ empirical quantile of the conformal score $s_1,\ldots ,s_n$, where $\lceil \cdot \rceil$ is a rounding function. The specific implementation process is to arrange the conformal scores of each sample in the calibration set in ascending order: $s_1<\ldots <s_n$. Then, $\hat{q}=s_{(n+1)(1-\alpha )/n}$ was selected as the conformal threshold, and use the $\hat{q}$ value to generate a prediction set:

9 $$\begin{aligned} \mathbb {C}(x_i)=\left[ f(x_i)_{\alpha /2}-\hat{q},f(x_i)_{1 - \alpha /2}+\hat{q}\right] \end{aligned}$$

The confidence interval $\mathbb {C}(x_i)$ achieves coverage guarantee by increasing or decreasing the interval between quantiles by $\hat{q}$. When the calibration set data is distributed similarly to the test set data, the confidence interval will satisfy the coverage characteristic in Eq. 7 on the test set. Two different error rates (non-conformity measure $\alpha$), 0.05 and 0.10, were selected to implement conformal prediction, and the confidence intervals covered the reference BP with 95% and 90% probability, respectively. Then, the confidence intervals are applied to the test set. In theory, when the distribution of the calibration set data and the test set data is consistent, the coverage of the confidence interval in the test set will be able to reach the theoretical value.

In practical applications, researchers usually hope that the confidence interval generated by conformal prediction is as small as possible, and the coverage of reference values is as high as possible, but this is difficult to achieve simultaneously. Therefore, the incremental cost-effectiveness ratio (ICER)^25^ was chosen to evaluate the trade-off between the uncertainty interval length and the reference value coverage. ICER is often used in economics and public health to evaluate the cost-effectiveness of two interventions. The calculation formula is as follows:

10 $$\begin{aligned} ICER=\frac{\Delta Cost}{\Delta Benefit} \end{aligned}$$

where $\Delta Cost$ represents the cost difference between the two interventions, and $\Delta Benefit$ represents the benefit difference between the two interventions (such as quality-adjusted life years, etc.). For conformal prediction, when different error rates $\alpha$ are selected, the corresponding cost changes are expressed as changes in interval length, and the corresponding benefit changes are expressed as changes in reference coverage. Therefore, in this study, ICER can be re-expressed as:

11 $$\begin{aligned} ICER=\frac{\Delta \hat{q}}{\Delta Coverage}=\frac{\hat{q}_{0.05}-\hat{q}_{0.10}}{Coverage_{0.05}-Coverage_{0.10}} \end{aligned}$$

An ICER of 1.25 means that for every 1% increase in coverage, an increase of 1.25 mmHg in interval length is required. When applied clinically, clinicians may be able to determine whether to achieve a higher reference coverage based on the ICER value.

### Validation and performance evaluation

To avoid the randomness of data set division affecting the model test results, this study adopted a rigorous data division strategy of 5-fold cross-validation^26^. Each time, 60% of the subject data were selected as the training set for training the GBRT model; 24% (40% $*$ 60%, that is, 60% of the remaining 40% of the subjects) of the subject data were selected as the calibration set for conformal prediction, and finally 16% (40% $*$ 40%, that is, 40% of the remaining 40% of the subjects) of the subject data were used as the test set to verify the model performance. The data set was randomly divided five times according to the division strategy described above, and the prediction results of the five test sets were averaged to evaluate the model performance.

The estimation performance of the GBRT model for BP estimation is evaluated according to the standards from IEEE 1708^27^ and the Association for the Advancement of Medical Instrumentation (AAMI)^28^. These two standards select the mean absolute difference (MAD), mean error (ME), and SD of error as the error evaluation coefficients, which can be calculated as:

12 $$\begin{aligned} MAD&=\frac{1}{N}\sum _{i=1}^N |(Y_{est_i}-Y_{ref_i})|\end{aligned}$$

13 $$\begin{aligned} ME&=\frac{1}{N}\sum _{i=1}^N (Y_{est_i}-Y_{ref_i}) \end{aligned}$$

14 $$\begin{aligned} SD&=\sqrt{\frac{1}{N-1}\sum _{i=1}^N (Y_{est_i}-Y_{ref_i}-ME)^2} \end{aligned}$$

where the $Y_{est_i}$ represents the model prediction for the *i*-th cardiac cycle, $Y_{ref_i}$ represents the reference BP value and *N* is the number of samples. The IEEE 1708 standard classifies wearable devices into four levels based on their estimation accuracy: “Grade A”, “Grade B”, “Grade C” and “Grade D”. For “Grade A” devices, the MAD between the wearable device and the reference BP should be less than 5 mmHg. The AAMI standard requires the ME and SD of BP estimation, i.e., ME ± SD $\le$ 5 ± 8 mmHg. Both standards specify the number of subjects required for validating device performance, and the amount of data used in our experiments complies with the requirements. Additionally, the scatter plot and Bland-Altman plot were utilized as a qualitative way to evaluate the correlation and agreement between the estimated BP by the proposed GBRT-based method and the reference BP. A t-test was also performed on the residuals between the estimation BP and the reference BP. In ambulatory settings, the BP distribution of the subjects has a large variability due to changes in their physiological state and daily activities. The coefficient of variability (CV)^29^ was introduced to measure the degree of variability of BP, and it can be calculated as:

15 $$\begin{aligned} CV=\frac{\epsilon }{\mu } \end{aligned}$$

where, $\epsilon$ is the standard deviation and $\mu$ is the mean. CV can measure the relative dispersion of the ambulatory BP data sets. Larger variability will affect the predictive performance of the BP estimation model, while the introduction of confidence intervals can provide reliable BP predictions even with high variability.

## Results

**Fig. 3:**
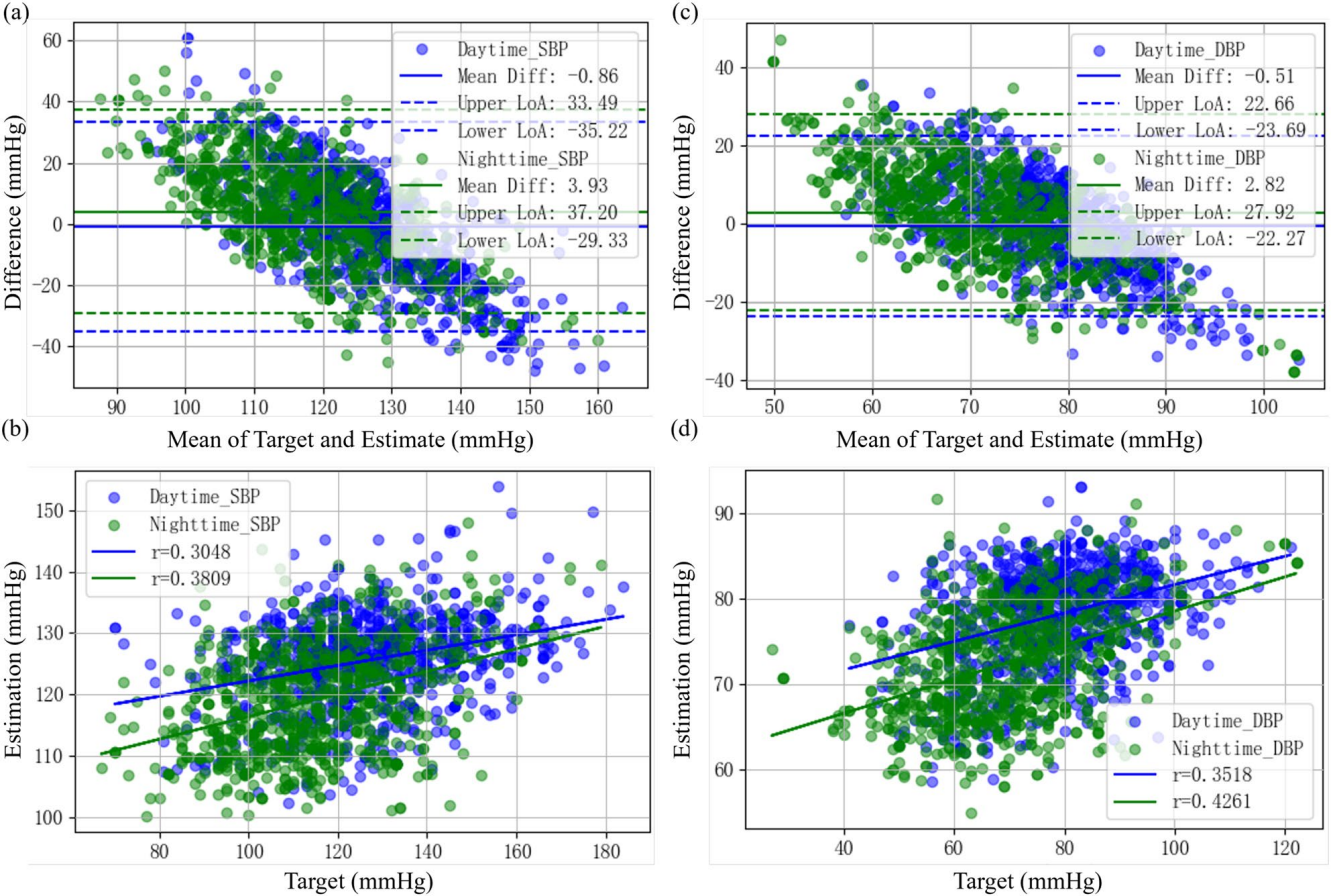
Bland-Altman plot and Scatter plot of the estimated 24 h ambulatory systolic blood pressure (SBP) (**a**), (**b**) and diastolic blood pressure (DBP) (**c**), (**d**) (blue for daytime measurements, and green for nighttime measurements) by the Gradient Boosting Regression Tree (GBRT) model versus reference SBP and DBP.

One of the five cross-validations was selected as a representative to evaluate the agreement between the estimated BP by the model and the reference BP. Figure 3a and c shows the Bland-Altman plot of the 24-h ambulatory SBP and DBP estimated by the proposed model and the reference SBP and DBP. It can be seen from the figure that most of the errors fall within the limits of agreement, and the same trend is observed for DBP. In addition, as shown in the scatter plot of Fig. 3b and d, for the two stages, the Pearson correlation coefficient between the estimated SBP and reference SBP are 0.3048 and 0.3809, respectively, and the Pearson correlation coefficients between the estimated DBP and the reference DBP are 0.3518 and 0.4261, respectively.

**Fig. 4:**
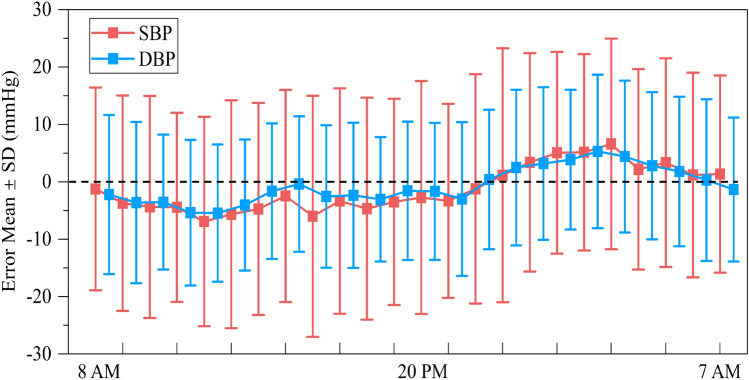
Mean ± standard deviation of hourly error of 24-h ambulatory blood pressure (BP) estimation.

Tables 3 and 4 show the performance of the GBRT model on the BP estimation of the test set in 5-fold cross-validation. Forsurements during the day, the average MAD in SBP and DBP estimated from the five experiments was 14.32 mmHg and 9.53 mmHg, respectively, with the mean and standard deviation of the errors (ME ± SD) are −3.14 ± 17.88 mmHg and −1.69 ± 11.76 mmHg, respectively. While for the nighttime measurements, the mean MAD of the SBP and DBP estimations are 14.22 mmHg and 10.13 mmHg, respectively, with average ME ± SD of the errors are 2.76 ± 17.52 mmHg and 2.83 ± 12.35 mmHg, respectively. It can be seen that the performance for the daytime and nighttime estimations is comparable. In addition, the performance of the 24-h BP estimate for each fold was also calculated by integrating the data from the daytime and nighttime phases. On average, the MAD of the 24-h SBP and DBP estimated from the five experiments were 14.27 mmHg and 9.83 mmHg, respectively, and the mean ME ± SD were -0.19 ± 17.95 mmHg and 0.57 ± 12.28 mmHg, respectively. However, DBP estimation consistently outperforms SBP estimation, aligning with findings from prior cuffless BP studies^21,30^. This discrepancy may be attributed to DBP’s greater physiological stability, as evidenced by its significantly lower variability (SD of DBP: 14.47 mmHg vs. SBP: 20.04 mmHg; see Fig. 2). As shown in Fig. 4, a representative fold in the 5-fold cross-validation was selected to plot the error graph of the mean and SD of the hourly error of its 24-h ambulatory BP measurement. For measurements in the daytime phase, the bias of the estimation error was below 0, while the bias of the error in the nighttime phase was generally greater than 0, but the SD of the error during the day and night was comparable. This is consistent with what is shown in the Table 4.

**Table 3:** Mean absolute difference (MAD) of blood pressure (BP) estimated by the gradient boosted regression tree (GBRT) model in the test set of 5-fold cross validation.

		MAD (mmHg)					
		1-Fold	2-Fold	3-Fold	4-Fold	5-Fold	Average
SBP	Daytime	15.21	13.08	13.88	15.05	14.40	14.32*
	Nighttime	15.05	14.00	14.10	13.82	14.12	14.22*
	24-h	15.13	13.54	13.98	14.43	14.25	14.27*
DBP	Daytime	10.10	8.49	9.33	9.79	9.92	9.53*
	Nighttime	10.69	9.78	10.43	9.60	10.18	10.13*
	24-h	10.40	9.14	9.88	9.70	10.05	9.83*

$*$ indicates statistical significance at the 0.05 level. Significant values are in bold.

**Table 4:** Mean error ± Standard Deviation (ME ± SD) of blood pressure (BP) estimated by the gradient boosted regression tree (GBRT) model in the test set of 5-fold cross validation.

		ME ± SD					
		1-Fold	2-Fold	3-Fold	4-Fold	5-Fold	Average
SBP	Daytime	− 4.23 ± 18.73	0.30 ± 16.23	− 0.86 ± 17.52	− 6.79 ± 18.94	− 4.12 ± 17.98	**− 3.14** ± **17.88**
	Nighttime	1.86 ± 18.66	5.60 ± 16.37	3.94 ± 16.95	0.09 ± 18.08	2.32 ± 17.55	**2.76** ± **17.52**
	24-h	− 1.19 ± 18.95	2.95 ± 16.51	1.54 ± 17.41	− 3.35 ± 18.83	− 0.9 ± 18.06	**− 0.19** ± **17.95**
DBP	Daytime	− 2.97 ± 12.31	0.35 ± 10.63	− 0.51 ± 11.82	− 3.88 ± 11.84	− 1.45 ± 12.23	**− 1.69** ± **11.76**
	Nighttime	1.54 ± 13.13	5.14 ± 11.16	2.81 ± 12.79	1.83 ± 12.23	2.82 ± 12.42	**2.83** ± **12.35**
	24-h	− 0.72 ± 12.92	2.75 ± 11.16	1.15 ± 12.42	− 1.03 ± 12.38	0.69 ± 12.51	**0.57** ± **12.28**

**Fig. 5:**
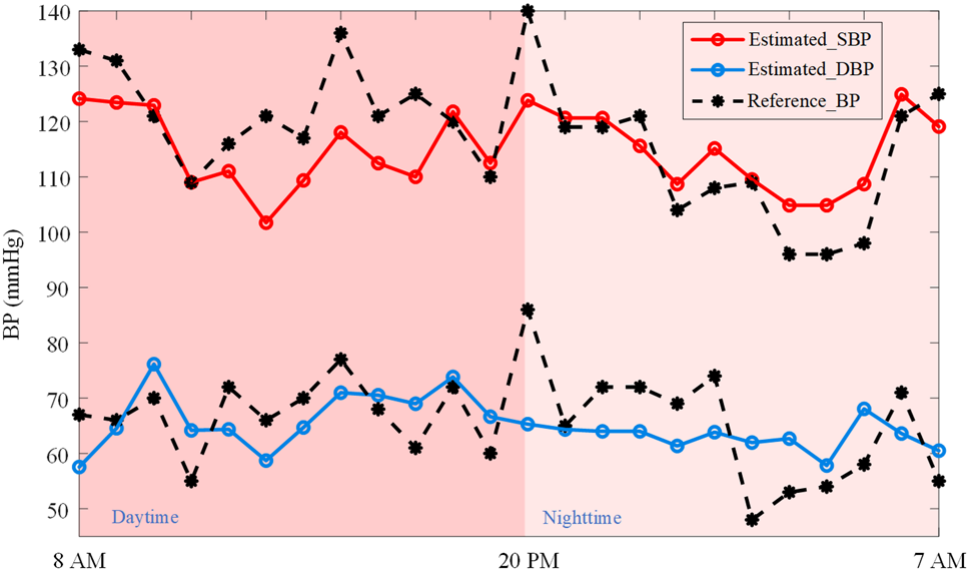
Estimated systolic blood pressure (SBP) (red) and diastolic BP (DBP) (blue) versus the reference BP (black) for one subject over 24 h.

**Fig. 6:**
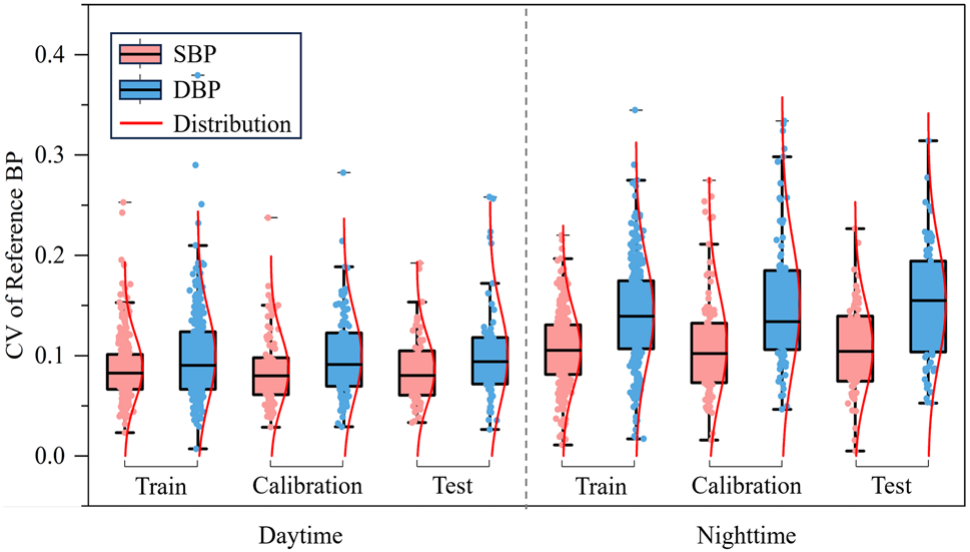
Coefficient of variability (CV) of reference systolic blood pressure (SBP) (pink) and diastolic blood pressure (DBP) (blue) for the training, calibration, and test dataset.

The estimated 24-h ambulatory BP against reference BP for one representative subject was further depicted in Fig. 5. It can be seen that the GBRT model can track the changes in 24-h ambulatory BP, and the changes in nighttime BP are greater than those in the daytime. Fig. 6 shows the box plots of CV of the reference BP for the training, calibration, and test dataset. For daytime and nighttime BP measurements, the distribution of the CV of the reference BP in the training, calibration, and test datasets is much the same, which further verifies the balance of the dataset division in this study. Of note, the median and distribution range of the CV of either SBP or DBP during the nighttime are significantly larger than those in the daytime stage.

The rate of the confidence intervals covering the reference BP value for the dataset was listed in Table 5. With a specified conformal error rate $\alpha$, the conformal threshold $\hat{q}$, i.e., the confidence interval, was obtained with the conformal prediction from the calibration dataset. Theoretically, when the conformal error rate $\alpha$ is 5% and 0.10, the coverage of the confidence interval with the reference value should be above 95%. When the error rate is 10%, the confidence interval coverage with the reference value should be above 90%. It can be seen from Table 5 that the conformal interval $\hat{q}$ for nighttime measurements is generally larger than that of the daytime measurements. This is potentially due to the larger BP variability at night than during the day, which results in a larger $\hat{q}$ value to achieve a coverage guarantee for the confidence interval. The coverage rate of the confidence interval for DBP was slightly above the theoretical value of conformal prediction, greater than 95% or greater than 90%. In comparison, the coverage rate for SBP during the daytime is slightly lower than the theoretical value, which may be related to the lower performance of the GBRT model for SBP estimation. However, the overall test set coverage distribution is consistent with the calibration set coverage distribution, which further verifies the consistency of the calibration set and test set data distribution, as well as the rationality of conformal prediction for generating confidence intervals. To evaluate the balance between the conformal threshold *q* and the reference value coverage, ICER was selected to compare the costs and effects of different conformal error rates. As shown in the rightmost column of the Table 5, when ICER is 1.25, it means that to increase the reference value coverage by 1%, the uncertainty interval length needs to be increased by 2.5 (1.25*2) mmHg. ICER can serve as a good indicator of the relationship between the $\hat{q}$ value and coverage. At the same time, when applied in clinical practice, clinicians may be able to determine whether to set a higher reference value coverage based on the ICER value, that is, whether to change the value of $\alpha$ in the conformal prediction. ICER provides clinician-in-the-loop systems with the flexibility of human expert feedback to improve clinical decision-making.

**Table 5:** Coverage rate of confidence intervals (CIs) on reference systolic blood pressure (SBP) and diastolic blood pressure (DBP).

	Phase	$\alpha$	$\hat{q}$ (mmHg)	Coverage ($\%$)	ICER
SBP	Daytime	0.05	8.39	94.67	1.75
		0.10	1.41	89.09	
	Nighttime	0.05	10.19	96.60	1.20
		0.10	4.34	91.72	
DBP	Daytime	0.05	5.85	95.55	0.95
		0.10	1.15	90.60	
	Nighttime	0.05	7.31	95.49	1.04
		0.10	3.32	91.65	

**Fig. 7:**
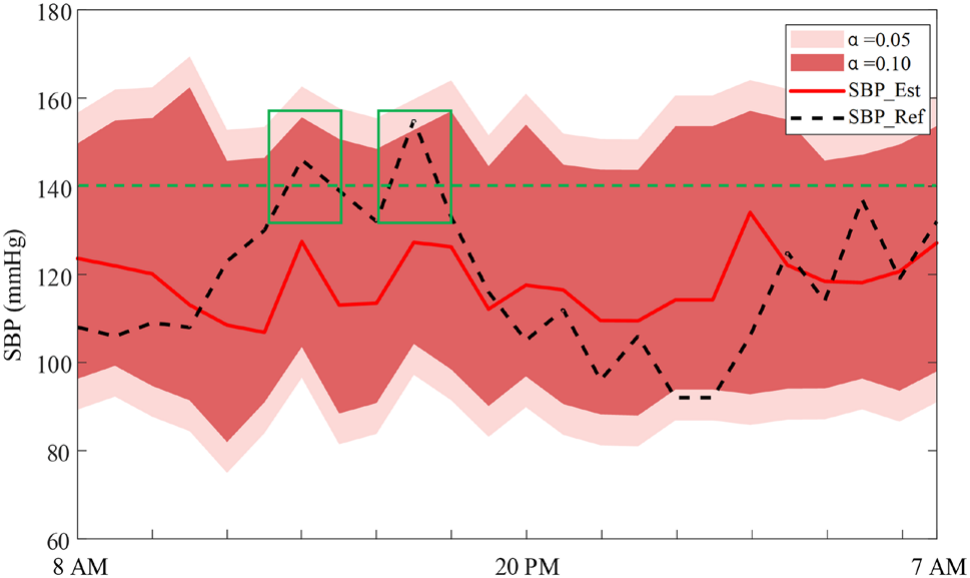
Estimated systolic blood pressure (SBP) (red line) and its confidence interval (dark red shade area for confidence level of 90%, and light red shade area for confidence level of 95%) versus reference SBP (black dot line).

The confidence intervals of estimated BP can be calculated with the combination of quantile regression and conformal prediction. Fig. 7 shows an example of the estimated SBP as well as the confidence interval of SBP estimation. The black dashed line represents the reference SBP; the red solid line represents the estimated SBP; the two-color shaded areas represent the confidence interval generated according to different error rate settings ($\alpha$=0.05/0.10), and the green dashed line represents the hypertension limit. According to the definition of hypertension published by the WHO in 2023, a subject can be diagnosed with hypertension if their SBP $\ge$ 140 mmHg or DBP $\ge$ 90 mmHg on two different days. The green box in Fig. 7 shows that when the BP predicted by the model is lower than the hypertension threshold, and the actual reference BP is higher than the hypertension threshold, the confidence interval can still consistently capture the hypertension condition.

The performance of the proposed method for 24-h BP estimation was compared with recent studies on ambulatory BP measurement and those based on the Aurora-BP dataset (Table 6). Hae et al.^31^ focused on the 24-h ABPM mean BP of patients receiving hypertension medication to assist in treatment decisions. Baseline clinical characteristics and 24-h ABPM data were fed into a CatBoost model to predict follow-up 24-h and daytime mean BP, however, hour-by-h BP fluctuations were not considered. Another study by Liu et al.^32^ reported a handcrafted feature-guided CNN and transformer network (HGCTNet) to address wearable ECG and PPG signals’ redundancy and global time dependency to improve cuffless BP estimation. The study used the full data of in-clinic and ambulatory measurements in the Aurora-BP dataset to validate the model. The results showed that the proposed CNN-Transformer model had a mean absolute error of 6.10 mmHg and 5.20 mmHg for SBP and DBP estimation, respectively. The accuracy of our method in estimating BP is not as good as that reported by Liu et al. The main reason is that this study only used ambulatory BP measurements to train and validate the model, and the variability of BP is greater than under controlled conditions. In addition, another study by Cisnal et al.^33^ estimated BP based on PPG signals and demographic characteristics with multiple regression models with validation on ambulatory measurement data from the oscillometric protocol of the Aurora-BP dataset. The results showed that the gradient boosting (GB) based method performed the best, with a mean absolute error of 11.35 mmHg and 7.85 mmHg for SBP and DBP estimation, respectively. The estimation performance of the ambulatory model was significantly lower than that of the in-clinic measurement data model, which also proves the challenge of ambulatory BP measurement. However, the above studies did not focus on the uncertainty quantification of ambulatory measurement and failed to provide reliable confidence intervals for BP prediction.

**Table 6:** Comparison between the proposed method and existing methods.

	Sample size	Training set (Model Input; Model Output)	Model	BP	Error metrics (mmHg)		UQ	CIs
					MAD	ME ± SD		
Hae et al.^31^	1129	Baseline clinical characteristics and 24-h ABPM; Follow-up mean BP	CatBoost	SBP	8.30	8.40 ± 7.00	–	–
				DBP	5.30	5.30 ± 4.30	–	–
Liu et al.^32^	1125	ECG, PPG and PPW signals; ABPM	HGCTNet	SBP	6.10	− 0.40 ± 8.60	–	–
				DBP	5.20	− 0.40 ± 7.00	–	–
Cisnal et al.^33^	500	PPG features; ABPM	GB	SBP	11.35	− 0.33 ± 14.54	–	–
				DBP	7.85	− 0.22 ± 10.10	–	–
This work	483	ECG and PPG features; ABPM	GBRT	SBP	14.27	− 0.19 ± 17.95	$\checkmark$	$\checkmark$
				DBP	9.83	0.57 ± 12.28	$\checkmark$	$\checkmark$

**Fig. 8:**
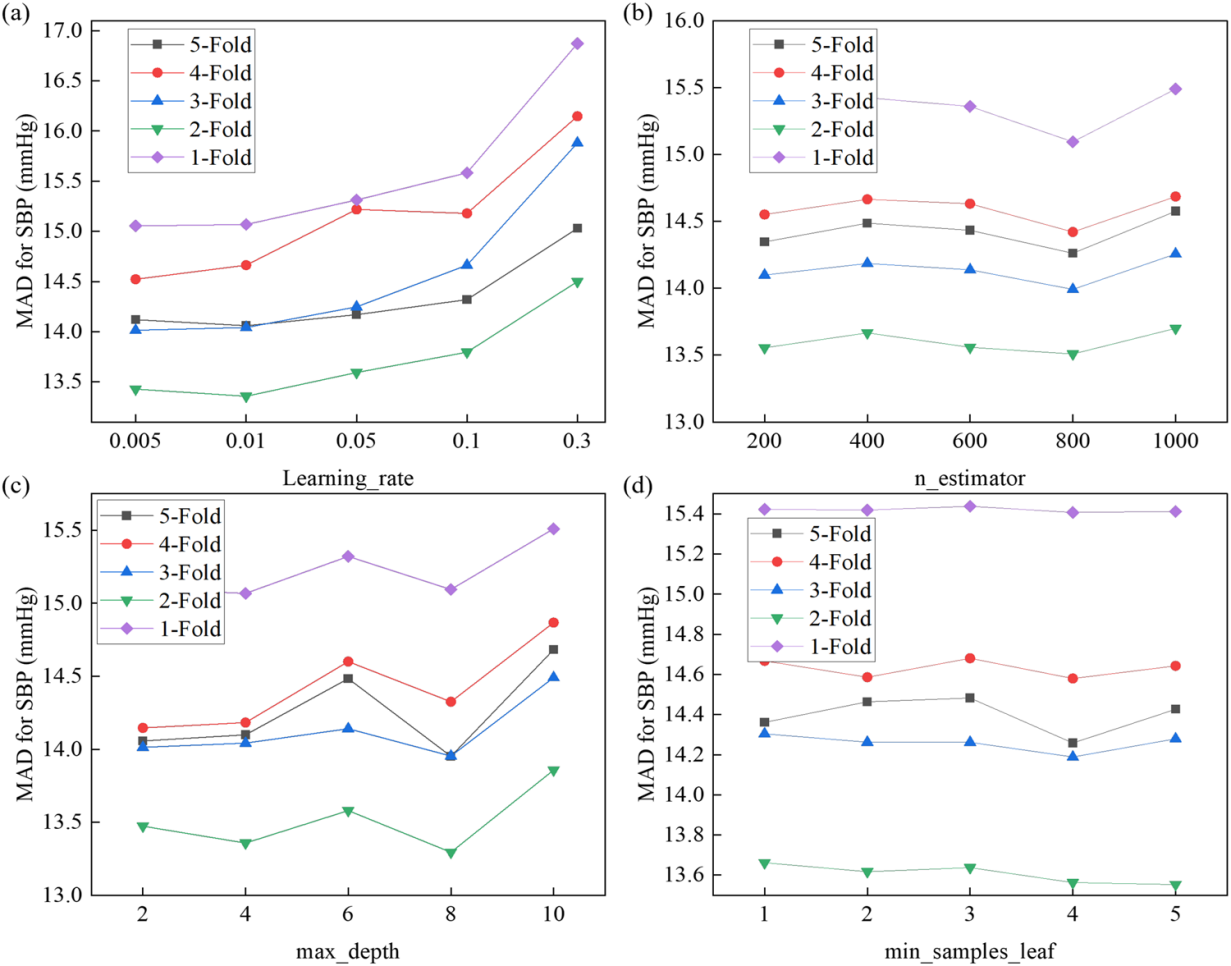
Blood pressure estimation performance with different hyperparameters: (**a**) learning rate, (**b**) number of regression trees, (**c**) maximum depth of the tree, and (**d**) minimum number of leaf nodes in the tree.

The hyperparameters of the GBRT model will affect the prediction performance of the model. Taking SBP as an example, this study uses the grid search method to determine the optimal learning rate, the number of regression trees, the maximum depth of the regression tree, and the minimum number of leaf nodes of the regression tree. Fig. 8 shows the results of all test sets in the 5-fold cross validation. When the learning rate is set to 0.01, the model achieves minimum MAD for SBP estimation. Although the estimated performance is not much different from the learning rate of 0.005, a larger learning rate helps the model converge faster. As seen from Fig. 8b, when the number of basic regression trees is greater than 400, the model estimation error gradually decreases, and increases after exceeding 800, which may be at risk of overfitting. In addition, when the maximum depth is 8, except for the fourth fold, all other cross experiments achieve the minimum MAD here. However, the model estimation performance does not change significantly with the change of the minimum number of leaf nodes. Still, the error is the smallest when the minimum number of leaf nodes is 4. Based on the above findings, lr = 0.01, n_estimator = 800, max_depth = 8, and min_samples_leaf = 4 were selected as the optimal hyperparameter values of the GBRT model.

## Discussion

To the best of our knowledge, this is the first study to quantify the uncertainty in ambulatory cuffless BP estimates and predict confidence intervals. The introduction of confidence intervals will improve the poor dynamic tracking of the BP estimation model and provide more reliable predictions for BP. Banerji et al.^34^ proposed that artificial intelligence tools used in clinical practice need to convey the uncertainty of the prediction while maximizing the accuracy of predictions. Uncertainty helps clinicians deepen their understanding of model predictions and further improve patient care. Vilde et al.^35^ proposed a time series prediction model based on conformal quantile regression to provide effective coverage prediction intervals for non-stationary and heteroscedastic time series. Experimental data results show that conformal quantile regression combined with traditional machine learning algorithms can provide confidence intervals with higher coverage and perform well in heteroscedastic data sets. However, no research has yet applied the common quantile regression algorithm to 24-h ambulatory BP measurement.

This study combines the lightweight GBRT model with conformal quantile regression to produce statistically rigorous confidence intervals. A single GBRT model tends to produce overconfident and invalid confidence intervals, with many times exceeding the boundary. The results in Table 4 shows that the confidence intervals after conformal prediction calibration has a high reference BP coverage in the test set. The overall test set coverage distribution is consistent with the calibration set coverage distribution, which further verifies the consistency of the calibration set and test set data distribution, as well as the rationality of the confidence interval generated by conformal prediction. When wearable BP measurement devices are applied to hypertension risk warnings, confidence intervals with higher confidence can enhance the trust of doctors and patients in the prediction results. However, from the distribution of estimated BP and reference BP drawn in the scatter plot in Fig. 3, it can be seen that the proposed GBRT model still has difficulties in tracking the highly changing BP conditions in an ambulatory environment. Therefore, the accuracy of model estimation is still affected by the patient’s daily activities, resulting in deviations in the measurement data, and is also limited by the impact of BP changes such as nighttime BP drops and drug treatment.

This study has some limitations. First, only data from daily ambulatory measurements were used to demonstrate the idea of quantifying cuffless BP with certainty. However, other scenarios with dynamic BP variations, such as continuous BP under drug therapy, were not implemented for verification. Second, the accuracy of the BP estimation is limited, and feature selection did not involve demographic information (e.g. age, BMI, and sex) and other more relevant features of arterial BP changes. Third, conformal prediction was implemented on all the subjects, resulting in estimated uncertainty intervals of fixed length. The quasi-conditional conformal prediction was not implemented to adapt to different subject datasets. Future studies should explore robust and accurate BP estimation with a better-performed BP estimation model with the consideration of conditional conformal prediction and validation on more comprehensive datasets.

## Conclusion

This study verified the feasibility and effectiveness of uncertainty quantification and confidence intervals estimation of ambulatory cuffless BP measurement. The BP estimation model trained only with ambulatory data is more suitable for people to measure BP anytime and anywhere during daily activities. The predicted CIs can provide a guaranteed reference BP coverage, which offers superior reliability compared to conventional single-point estimates that are inherently limited by measurement errors and model uncertainties. The introduction of this research method can improve the trust of doctors and patients in wearable devices and enhance their confidence in decision-making.

## Data Availability

The datasets used and/or availability during the current study are available from the corresponding author on reasonable request.
